# Boat noise prevents soundscape-based habitat selection by coral planulae

**DOI:** 10.1038/s41598-018-27674-w

**Published:** 2018-06-18

**Authors:** David Lecchini, Frédéric Bertucci, Camille Gache, Adam Khalife, Marc Besson, Natacha Roux, Cecile Berthe, Shubha Singh, Eric Parmentier, Maggy M. Nugues, Rohan M. Brooker, Danielle L. Dixson, Laetitia Hédouin

**Affiliations:** 1 EPHE, PSL Research University, UPVD-CNRS, USR3278 CRIOBE, 98729 Moorea, French Polynesia; 2 Laboratoire d’Excellence “CORAIL”, Moorea, French Polynesia; 3 Laboratoire de Morphologie Fonctionnelle et Evolutive, Université de Liège, B-4000 Liège, Belgium; 4 Institut de Génomique Fonctionnelle de Lyon, Université de Lyon; Université Lyon 1; CNRS UMR 5242, Ecole Normale Supérieure de Lyon, 46 allée d’Italie, 69364 Lyon, France; 5 Observatoire Océanologique de Banyuls-sur-Mer, UMR7232, Université Pierre et Marie Curie Paris, 1 avenue du Fontaulé, 66650 Banyuls-sur-Mer, France; 6 University of the South Pacific, School of Marine Science, Suva, Fiji; 7 Institut des Récifs Coralliens du Pacifique, IRCP, Moorea, French Polynesia; 8 School of Marine Science and Policy, University of Delaware, Robinson Hall, Newark, DE 19716 USA

## Abstract

Understanding the relationship between coral reef condition and recruitment potential is vital for the development of effective management strategies that maintain coral cover and biodiversity. Coral larvae (planulae) have been shown to use certain sensory cues to orient towards settlement habitats (*e.g*. the odour of live crustose coralline algae - CCA). However, the influence of auditory cues on coral recruitment, and any effect of anthropogenic noise on this process, remain largely unknown. Here, we determined the effect of protected reef (MPA), exploited reef (non-MPA) soundscapes, and a source of anthropogenic noise (boat) on the habitat preference for live CCA over dead CCA in the planula of two common Indo-Pacific coral species (*Pocillopora damicornis* and *Acropora cytherea)*. Soundscapes from protected reefs significantly increased the phonotaxis of planulae of both species towards live CCA, especially when compared to boat noise. Boat noise playback prevented this preferential selection of live CCA as a settlement substrate. These results suggest that sources of anthropogenic noise such as motor boat can disrupt the settlement behaviours of coral planulae. Acoustic cues should be accounted for when developing management strategies aimed at maximizing larval recruitment to coral reefs.

## Introduction

Larval replenishment is essential for the maintenance and recovery of marine benthic communities, especially in systems that rely on functionally important or foundation species, such as coral reefs^1^. Worldwide, coral reefs are facing unprecedented acute and chronic threats due to anthropogenic impacts that are driving substantial coral mortality and habitat loss^2,3^. As corals are the primary ecosystem engineer within reef communities, providing habitat and resources for many reef-associated species, the continued recruitment of coral larvae (planulae) into habitats that are favourable to survival is vital for the reestablishment, recovery, and resilience of degraded reef ecosystems^1,4^. Evidence is mounting that coral planulae are active participants during dispersal and recruitment^4^. However, the sensory and behavioural mechanisms used to disperse and return to appropriate habitat remains poorly understood, especially in the context of rapid local and global environmental change^4,5^.

Once in the vicinity of a coral reef, settlement-stage planulae respond to a variety of environmental cues, including, but not limited to: light, colour, depth, pressure and waterborne chemicals^4,6,7^. Sensory cues produced by components of the benthic community can also influence coral settlement. For instance, species of crustose coralline algae (CCA) provide positive settlement cues for many coral species^8–10^ while algal turfs, macro algae and cyanobacteria generally produce negative cues^11,12^. This effect has been attributed to physical, chemical, and microbial mediated mechanisms, including the release of primary and secondary metabolites by algae^4,13^. In the context of local and global threats to coral reefs, it has been suggested that the chemically-mediated responses of planulae could further reduce the resilience of degraded reefs if these systems are producing repellent chemical cues (*e.g*. from macro algae)^14^. In addition, global stressors, such as ocean acidification and elevated sea surface temperatures, can also reduce coral settlement, most likely due to the disruption of chemically-mediated mechanisms between coral planulae and CCA^15,16^.

Acoustic cues are also used by many marine organisms to gather information on the location of mates or resources, orient towards habitats, when interacting with conspecifics, and detect predators or prey^17,18^. On coral reefs, noise (different from pressure amplitude) is highest closest to the reef due to a high density of different sound sources (*e.g*. breaking waves, snapping shrimps, and soniferous fishes). Reef’s sound is temporally variable at a range of scales, changing diurnally, over lunar cycles, and also with the seasons^17–19^. These acoustic cues play an important role during recruitment with several studies showing the phonotaxis of larval fishes and crustacean to reef habitats^5^. While coral planulae lack structures analogous to the ‘ears’ of vertebrates such as fishes, they are densely covered with exterior cilia, which may allow them to sense and react to underwater sound fields. For example, coral planulae respond positively to the natural reef sounds produced by resident fish and crustaceans^20^ and ‘high amplitude’ soundscapes have been shown to generate a higher settlement rates compared to quieter environments^21^. However, further investigation is needed to definitively understand the mechanisms underlying the perception of positive and negative acoustic cues on the ability of coral planulae to find an appropriate reef substrate, especially CCA.

Anthropogenic noise is now considered a global pollutant. In coastal marine environments, a major source of this noise is boat traffic. Boat noise can impact the behaviour (*e.g*. acoustic communication, antipredator defence, foraging) and physiology (*e.g*. ventilation rates, metabolic rates, heart rates) of marine organisms from a broad range of taxa^22,23^. For example, boat noise can disrupt the responses of fish larvae to natural reef sounds^24^ with potential implications for habitat selection, population dynamics, and the resilience of coral reef habitats near human development^24^. Additionally, despite the importance of Marine Protected Areas (MPA) in the protection and conservation of marine species and resources^25,26^, whether the different soundscapes produced by MPAs and non-MPAs affect larval settlement remains untested^5^. Healthy coral reef communities are associated with specific ambient soundscapes^27,28^ with a positive correlation between coral cover, fish density and sound levels^29^. This may be a result of positive feedback mechanisms, with healthy, biodiverse reefs inherently louder and richer acoustically than degraded locations^30,31^.

Here, we investigated the effect of different soundscapes (recorded from protected and unprotected reefs) on the phonotaxis of coral planulae (both *Pocillopora damicornis* and *Acropora cytherea*) towards live CCA substrate and tested the potentially negative effect of an anthropogenic sound source (boat noise) on the preference for live CCA over dead CCA substrate at Moorea island. Specifically, we examined if 1) coral planulae orient more frequently towards live CCA in acoustically “un-disturbed” seawater (control). We then determined if 2) cues present in protected reef (3 MPA sites) soundscapes increases the number of planulae settling on live CCA compared to soundscapes of unprotected reefs (3 non-MPA sites). Finally, we examined if 3) anthropogenic (boat) noise affected larval settlement patterns.

## Results

**Figure 1:**
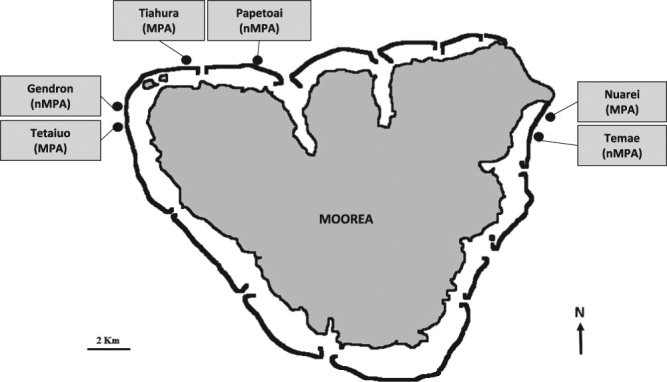
Map of recording sites in three Marine Protected Areas (MPA) and three non-Marine Protected Areas (non-MPA). Map drawn by the authors from an aerial photograph of Moorea taken by the CRIOBE in 2008 from a private plane.

The sounds of 3 MPAs, 3 adjacent non-MPA sites, and repeated boat passes were recorded at Moorea Island (Fig. 1). Together with a control (no sound) treatment, all sounds were tested on *P. damicornis*, while the sounds of only one MPA/non-MPA pair, and the boat sound were tested on *A. cytherea*.

### Sound analyses

**Figure 2:**
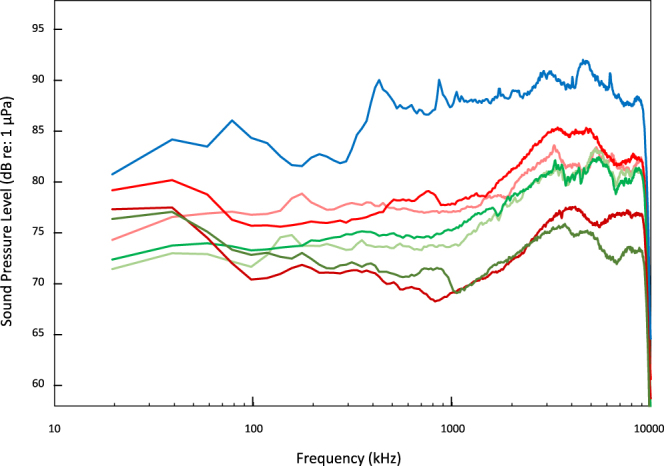
Average power spectra (Sound Pressure Levels, dB re: 1 μPa) of the sounds played to coral planulae. Boat noise is represented in blue, MPA sounds are represented in red (Tetaiuo: light red, Tiahura: red and Nuarei: dark red) and non-MPA sounds are represented in green (Gendron: light green, Papetoai: green and Temae: dark green).

Sound samples mostly differed in frequencies above 0.3 kHz (Fig. 2). The boat sound sample exhibited an average sound pressure level (SPL) of 88 ± 3 dB re 1 μPa. Lower sound levels were found for frequencies under 0.3 kHz (<85 dB re 1 μPa). The highest SPL were found at three local maxima at 0.4 kHz, and 0.9 kHz (90 dB re 1 μPa) and at 5 kHz (93 dB re 1 μPa).

Sounds of Tetaiuo and Tiahura (MPAs) showed a similar pattern of sound levels with an average SPL of 78 ± 2 dB re 1 μPa up to 2 kHz, before an increase and local maxima of 83 dB re 1 μPa and 85 dB re 1 μPa respectively, at *ca*. 3.5 kHz. Their respective non-MPAs, Gendron and Papetoai, also showed similar spectra but with lower sound levels with an average SPL of 74 ± 1 dB re 1 μPa up to 2 kHz, before an increase and a common local maximum of 82 dB re 1 μPa at *ca*. 3.5 kHz. The last MPA/nMPA pair, Nuarei and Temae, had similar spectra with the lowest levels and an average SPL of 72 ± 3 dB re 1 μPa up to 1 kHz, before an increase and local maxima of 77 dB re 1 μPa and 75 dB re 1 μPa respectively, at *ca*. 4 kHz (Fig. 2).

Comparisons between MPAs and their adjacent non-MPAs revealed significant differences (Kruskal-Wallis, χ²_5_ = 16.5–140, P = 0.02 and P < 10^−3^ for day and night respectively) in the sound pressure levels in the low frequencies (20 Hz–2 kHz). Tetaiuo and Gendron (t = 3.54, P = 0.01) and Tiahura and Papetoai (t = 3.90, P < 0.005) only differed during night recordings (day, t = 0.02–0.69, P = 1). Nuarei and Temae did not differ (day, t = 1.17, P = 0.94; night, t = 1.91, P = 0.54). Significant differences also appeared in higher frequencies (2–20 kHz) (Kruskal-Wallis, χ²_5_ = 177, P < 10^−3^). Tetaiuo and Gendron showed significantly different sound intensities both during day (t = 3.69, P = 0.006) and night (t = 3.71, P = 0.006). Tiahura and Papetoai showed no differences during day (t = 0.58, P = 0.99) nor night recordings (t = 0.01, P = 1). Lastly, the Nuarei and Temae still displayed similar sound intensities between day and night (t = 0.25–0.89, P = 1) (Fig. 2).

### Choice experiments on Pocillopora damicornis

**Figure 3:**
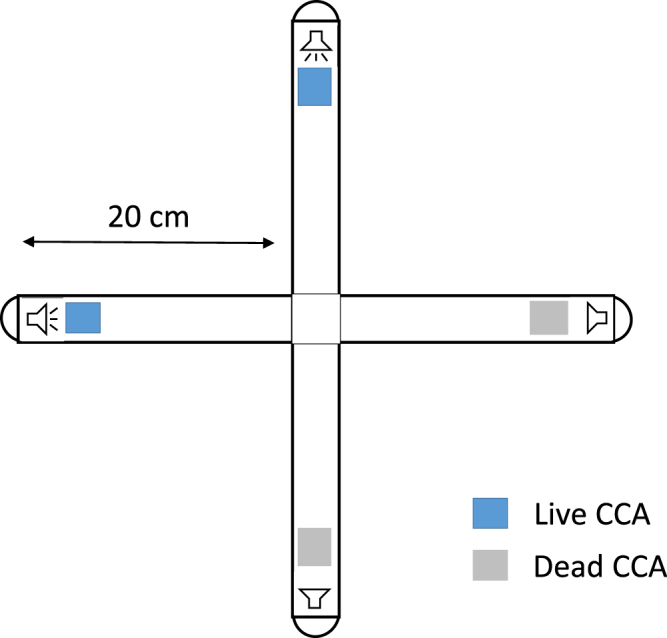
Schematic representation of the experimental apparatus used for choice experiments.

**Figure 4:**
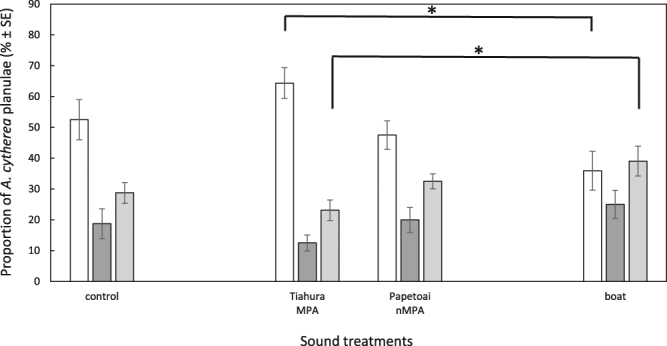
Distribution of *Pocillopora damicornis* planulae (% ±SE) in the choice chambers. Results at t = 4 h for the different sound treatments: no sound (control) (n = 13), Tetaiuo MPA sounds (n = 12), Gendron non-MPA sounds (n = 12), Tiahura MPA sounds (n = 12), Papetoai non-MPA sounds (n = 12), Nuarei MPA sounds (n = 12), Temae non-MPA sounds (n = 12) and boat noise (n = 13). Stars indicate significant differences in the proportion of planulae between sound treatments and the control condition in the live (white stars) and dead CCA arms (light grey star). Asterisks indicate significant differences in the proportion of planulae between MPAs and their respective non-MPA sound treatments at an alpha level of 0.05. Results are from a 2-way ANOVA followed by Tukey HSD post hoc tests. Live CCA arms are in white, dead CCA arms are in light grey, central compartments (no choice) are in dark grey.

In the no-noise control treatment, 39 ± 2% (mean ± SE) of *P. damicornis* planulae were present in arms containing live CCA while 26 ± 3% were in arms containing dead CCA (Figs 3 and 4). Planulae attraction towards live CCA was significantly affected by sound treatment (2-way ANOVA, F_3,98_ = 9.98, P < 10^−3^) with MPA sounds resulting in more planulae in the live CCA arms than any other sound treatment (Table 1, Fig. 4). When MPA sounds were played behind the live CCA substrate, 48 ± 6% of planulae preferred live CCA arms while 26 ± 4% still preferred dead CCA arms. In contrast, there were significantly more planulae in the dead CCA arms only when boat noise was played (2-way ANOVA, F_3,98_ = 4.97, P = 0.003) (Table 1). A higher proportion (43 ± 6%) of planulae were found within the chamber arms containing dead CCA than within live CCA chamber arms (24 ± 3%) (Fig. 4).

**Table 1:** P values of all pairwise comparisons (Tukey HSD post-hoc test) comparing the proportion of *Pocillopora damicornis* planulae between the 4 different treatments (control, MPAs, non-MPAs and boat) for the live CCA arm (white) and for the dead CCA arm (grey).

	control		MPAs		nMPAs	
	Live	Dead	Live	Dead	Live	Dead
MPAs	**0.045**	0.99				
nMPAs	0.18	0.44	**<10** ^**−3**^	0.28		
boat	**0.026**	**0.003**	**<10** ^**−3**^	**<10** ^**−3**^	0.18	**0.004**

Significant differences at an alpha level of 0.05 are in bold type.

When looking at the differential effects of sound type, a significant effect was found (2-way ANOVA, F_7,94_ = 5.30, P < 10^−3^). Playbacks of all MPA sounds attracted significantly more planulae towards live CCA than sounds of their respective non MPA (Table 2). Tetaiuo and Tiahura appeared as the most attractive stimuli with significantly more planulae attracted towards live CCA than in control condition (Table 2). Boat noise significantly decreased the number of attracted planulae compared to the 3 MPAs sounds and no sound (control) condition. The number of planulae found in the dead CCA arms was significantly higher when boat noise was played (ANOVA, F_7,94_ = 3.31, P = 0.003) compared to any other sound type (Table 2, Fig. 4).

**Table 2:** P values of all pairwise comparisons (Tukey HSD post-hoc test) comparing the proportion of *Pocillopora damicornis* planulae between the 8 different sound types (control, Tetaiuo MPA, Gendron non-MPA, Tiahura MPA, Papetoai non-MPA, Nuarei MPA, Temae non-MPA and boat) for the live CCA arm (white) and for the dead CCA arm (grey).

	control		MPA Tetaiuo		nMPA Gendron		MPA Tiahura		nMPA Papetoai		MPA Nuarei		nMPA Temae	
	Live	Dead	Live	Dead	Live	Dead	Live	Dead	Live	Dead	Live	Dead	Live	Dead
MPA Tetaiuo	**0.047**	0.79												
nMPA Gendron	0.53	0.36	**0.02**	0.24										
MPA Tiahura	**0.021**	0.91	0.57	0.69	**0.004**	0.44								
nMPA Papetoai	0.43	0.36	**0.012**	0.52	0.87	0.07	**0.003**	0.31						
MPA Nuarei	0.81	0.84	0.13	0.65	0.39	0.48	**0.04**	0.94	0.32	0.28				
nMPA Temae	0.061	0.07	**0.0004**	0.12	0.22	**0.008**	**<10** ^**−3**^	0.06	0.29	0.39	**0.04**	0.5		
boat	**0.024**	**0.003**	**<10** ^**−3**^	**0.007**	0.11	**<10** ^**−3**^	**<10** ^**−3**^	**0.002**	0.15	**0.04**	**0.014**	**0.002**	0.74	0.25

Significant differences at an alpha level of 0.05 are in bold type.

### Choice experiments on *Acropora cytherea*

**Figure 5:**
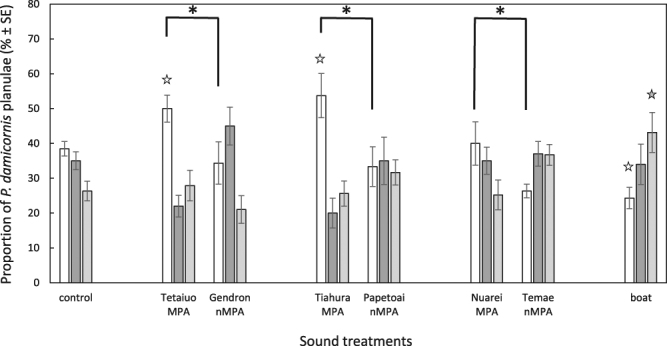
Distribution of *Acropora cytherea* planulae (% ± SE) in the choice chambers. Results at t = 4 h for the different sound treatments: no sound (control) (n = 8), Tiahura MPA sounds (n = 8), Papetoai non-MPA sounds (n = 8) and boat noise (n = 8). Asterisks indicate significant differences in the proportion of planulae in the live and dead CCA arms between Tiahura MPA and boat sound treatments at an alpha level of 0.05. Results are from a one-way ANOVA followed by Tukey HSD post hoc tests. Live CCA arms are in white, dead CCA arms are in light grey, central compartments (no choice) are in dark grey.

In the no-noise control treatment, 53 ± 7% of *A. cytherea* planulae were present in arms containing live CCA while 29 ± 3% were in arms containing dead CCA (Figs 3 and 5). Planulae attraction towards live CCA was significantly affected by sound treatment (One-way ANOVA, F_3,28_ = 4.32, P = 0.01). However, only MPA (Tiahura) sounds resulted in more planulae in the live CCA arms compared to boat noise (Table 3, Fig. 5). When MPA sounds were played behind the live CCA, 64 ± 5% of planulae preferred the live CCA arms while 36 ± 6% preferred the live CCA arms when boat noise was played back. In contrast, in the dead CCA arms (One-way ANOVA, F_3,28_ = 3.46, P = 0.03), the number of planulae was significantly higher when boat sounds were played behind live CCAs compared to MPA sounds (Table 3, Fig. 5).

**Table 3:** P values of all pairwise comparisons (Tukey HSD post-hoc test) comparing the proportion of *Acropora cytherea* planulae between the 4 different sound types (control, Tiahura MPA, Papetoai non-MPA and boat) for the live CCA arm (white) and for the dead CCA arm (grey).

	control		MPA Tiahura		nMPA Papetoai	
	Live	Dead	Live	Dead	Live	Dead
MPA Tiahura	0.46	0.69				
nMPA Papetoai	0.92	0.88	0.18	0.27		
boat	0.19	0.21	**0.007**	**0.02**	0.48	0.57

Significant differences at an alpha level of 0.05 are in bold type.

## Discussion

Coral planulae of both *P. damicornis* and *A. cytherea* displayed a significant preference for live CCA in the absence of acoustic cues. This attraction was likely driven by chemical cues released by the CCA and/or associated microbial films^8,10,32^. However, planulae seem to be affected by soundscapes and capable of distinguishing between sound sources. In particular, sounds produced by boats significantly reduced the attraction to the live CCA in *P. damicornis* when compared to the no-noise control. In *A. cytherea*, this reduction was significant when compared to MPA sound. Each of the sound treatments used (MPA, non-MPA, boat noise) had peculiar acoustic features that may have been used by coral planulae as orientation cues, similar to what has been observed in many other taxa^18,21,33^. The different areas of a reef are known to produce distinctive soundscapes^27,29,34,35^, with healthy locations producing significantly louder and richer acoustic signals than degraded sites or sites impacted by human activities^30,36^. Thus, the present study suggests that the soundscapes generated within MPAs may facilitate increased larval attraction. The sound records of MPA and non-MPA were collected within three MPA/adjacent non-MPA pairs with one pair from each coast of Moorea Island. Differences in acoustic features were present between two of the three MPA/ non-MPA pairs, with significantly higher sound levels within the MPA than within their respective adjacent non-MPA (Tetaiuo/Gendron and Tiahura/Papetoai). The last pair, Nuarei/Temae, still induced a different proportion of larvae in the live CCA arms though, even if this pair was the quietest with similar spectra between the MPA and its corresponding non-MPA. Overall, results highlight, for the first time, that free-swimming coral planulae are capable of using auditory cues for habitat discrimination and selection (Figs 2, 4 and 5) and the identification of what acoustic feature may trigger attraction towards a suitable habitat deserves to be investigated in the future.

Sound is characterized by the propagation of particle motion and acoustic pressure^33^. Measuring particle motion would have needed the use an accelerometer, which could not fit into the experimental setup. In fishes, some species can detect the particle motion component by means of external neuromasts along the lateral line, which are relatively similar to the cilia of coral planulae^20^. Other fishes can also detect acoustic pressure and translate it into particle motion by means of anatomical linkages between the gas-filled swim-bladder for instance and the otoliths located in their inner ear^17^. As suggested by Vermeij *et al*.^20^ and in absence of morpho-functional studies on cilia of planulae, we anticipate that coral planulae can only respond to particle motion^37^. This mechanism likely constrains their ability to perceive reef sounds to relatively short distances^37^. Nevertheless, determining the sensory mechanism that permits coral planula to distinguish between different sound recordings (*i.e*. propagation of particle motion, acoustic pressure, or sound levels) is essential for identifying the spatial scales over which noise cues are behaviourally relevant. It is also important to determine whether the observed responses are due to sound avoidance or rather an impaired ability to detect other stimuli.

Understanding global stressors impacting coral settlement has generated considerable attention^15,16^. To this end, this study identifies a new pollutant for coral: anthropogenic sound in the form of boat noise. Anthropogenic noise increasingly contributes to natural soundscapes on a global scale^38,39^, and may alter settlement patterns of marine fish larvae^5^. For example, boat noise reduced the attraction to reef sounds by 13% in settlement stage fish larvae^24^. Here, boat noise reduced the number of *P. damicornis* planulae attracted to live CCA too. In *A. cytherea*, boat noise diminished the attraction to live CCA to the extent that larvae avoided the preferential live CCAs. Therefore, boat traffic near coral reefs may require additional management during peak recruitment periods to increase the likelihood of coral larvae locating favourable substrate. Coral larval phonotaxis towards suitable habitats could be impaired by the noise caused by boat traffic which is predicted to increase, even in small South Pacific Islands, such as Moorea^40^. Finally, the negative response to boat noise suggests that coral larvae do not simply respond positively to elevated sound levels that would be easier to detect but that more subtle features of soundscapes may come into play and be used to orient during recruitment.

Overall, the mechanisms that facilitate larval behaviour and settlement are intricately linked to population replenishment, persistence, and resilience to ecological changes. As a result, understanding the processes that influence the sensory abilities of marine larvae is fundamental for effective marine management and conservation^41^. Far from being passive propagules, coral larvae use a combination of chemical, visual, physical, and auditory cues for fine-scale habitat selection during settlement processes^7,10,14,42^. Consequently, stressors that alter the sensory cues that larvae receive have the potential to impair recovery potential^43,44^. The present study suggests that anthropogenic noise may impact larval phonotaxis in two widespread and common coral species, while highlighting a potential interaction between acoustic and chemical cues on fine-scale habitat choice. Further information on key acoustic stimuli, such as local soniferous marine species, or boat noise characteristics, acting as either facilitators or inhibitors of larval recruitment in coral reefs is needed to enhance management initiatives and mitigate anthropogenic or natural disturbances.

## Methods

### Coral colonies and CCA

*Pocillopora damicornis* (L., 1758) is a brooding coral that releases competent planulae for 6–7 days, monthly, around the new moon, at Moorea^45^. *Acropora cytherea* (Dana, 1846) is a broadcast spawning coral that releases egg/sperm packages monthly (around new moon) between September and November. Planulae of *P. damicornis* and bundles (egg/sperm packages) of *A. cytherea* were collected by holding adult colonies (10 colonies of each species for each sampling month - diameter range: 15–25 cm; height range: 10–20 cm) removed from the northern lagoon of Moorea, French Polynesia in shaded outdoor aquaria with oxygenated running seawater (Temperature: 28.3 ± 0.2 °C, Salinity: 36 ppt). Planulae were collected using hand nets. Bundles were collected by hand using 50 mL plastic tubes, mixed in a 10 L container with 5 L of 5 μm filtered seawater, left for 2 h to allow gamete bundles to break apart and fertilization to occur. All embryos were then pipetted from the container and transported to gently aerated, 20 L containers for planulae development. Coral larvae were tested when active swimming and bottom searching behaviour was displayed 4 days post-fertilization.

Experiments on *P. damicornis* planulae were conducted from April to May 2016, September to November 2016 and February 2017 to acquire enough larvae replicates for sound treatments. The experiments on *A. cytherea* planulae were conducted between September and November 2016. Before each experiment, several pieces of CCA (*Porolithon onkodes*) were collected on reefs in Moorea’s northern coast and kept in the same conditions as coral colonies. Some CCA pieces were put in bleach for 12 hours and then washed in freshwater and seawater in order to obtain dead CCA fragments used as a substrate control. New pieces of live and dead CCA were used for each trial and within 24 h after their field collection.

### MPA and non-MPA sites

Recording sites were located on the north coast of Moorea Island (French Polynesia). Three MPA sites, *i.e*. Tetaiuo (S 17.50950°; W 149.92950°), Tiahura (S 17.48260°; W 149.87290°) and Nuarei (S 17.50114°; W 149.75495°) and 3 non-MPA sites located nearby, *i.e*. respectively Gendron (S 17.50131°; W 149.92755°), Papetoai (S 17.48257°; W 149.87290°) and Temae (S 17.51030°; W 149.76119°) were chosen (Fig. 1). The MPA sites have been no-take areas since 2004^36^. Benthic and fish surveys were conducted with 6 belt transects (25 × 4 m) to describe the coral and fish characteristics of all locations (data collected in April 2016 and available at www.observatoire.criobe.pf/). Live coral cover within the 6 sites ranged from 10 to 50%, fish density and species diversity ranged from 140 to 1055 fish, and from 33 to 55 species for 100 m² respectively, Shannon-Wiener index of diversity (H) ranging from 2.4 to 3.4 (for a more detailed description, see Bertucci *et al*.^36^).

In the present study, sounds of MPA vs. non-MPA were tested, however, the acoustic characteristics of each sound depend on the quality of habitat (e.g., % of live coral, number of species) and specific soniferous marine species in each MPA and each non-MPA. Therefore, MPAs and non-MPAs cannot be considered as absolute spatial replicates and the present study will not highlight that protection status favours or not the coral settlement, but will rather highlight that the general quality of an habitat, mediated by acoustic cues, could favour coral settlement (for a more detailed description of the 6 sites, see Bertucci *et al*.^36^).

### Sound records

Four sound treatments were examined: (a) no sound (control), (b) reef sound from MPAs, (c) reef sound from non-MPAs, and (d) boat noise.

MPAs and non-MPAs reef sounds were recorded using a hydrophone (HTI-96-MIN with inbuilt preamplifier; sensitivity −165 dB re 1 V/μPa; frequency range 2 Hz–30 kHz; High Tech Inc., Gulfport, MS) connected to a underwater Digital Spectrogram (DSG) Long-Term Acoustic Recorder (Loggerhead Instruments, Sarasota, FL, USA)^36^. A recorder was placed on the outer slope at 10 m depth in the MPA and non-MPA sites from April 1st to 3rd, 2016. Each recorder was programmed to record 5 minutes per hour over a 48-hr period. Recordings were digitized at 44.1 kHz (16-bit resolution). Visual and audio inspection of spectrograms revealed no boat noise in the recordings. Four-hour long MPA and non-MPA tracks were created by concatenating the 5-min files in chronological order. Power spectra of the sounds were calculated (Fast Fourier Transform FFT, 1024 points Hamming window, 75% overlap) and average sound pressure levels (Root Mean Square SPL, in dB re: 1 μPa) in the 20 Hz–2 kHz and in the 2–20 kHz frequency bands were compared with Kruskal-Wallis tests followed by Tukey’s post-hoc tests for pairwise comparisons in order to examine differences between MPAs and non-MPAs.

A total of 36 recordings of two outboard motorboats with 25-horse power Yamaha engines passes were made, with one boat per recording. Boats drove a 100 m-long straight line, starting 50 m away from the hydrophone and passed the hydrophone at a distance of 10 m. Boats were recorded during day at 10 m depth in a bay in the northern lagoon of Moorea (Opunohu bay) in April 2016, using a hydrophone (HTI- 96-MIN) connected to a solid-state recorder (Edirol R-09HR 16-bit recorder; sampling rate 44.1 kHz; Roland Systems Group, Bellingham, WA)^46^. Twelve without boat ambient-noise recordings were also made at the same recording location each day. The recorder was calibrated using pure sine wave signals synthesized in SAS Lab Pro version 5.2.07 software (Avisoft Bioacoustics, Glienicke, Germany), played on an MP3 player, and measured in line with an oscilloscope. Following the methodology described in Nedelec *et al*.^46^, boat recordings were clipped to 45 s samples representing one boat pass. Ambient-noise recordings were clipped into 64 s samples. Five minutes stretches were created by randomly mixing one boat pass and four ambient-noise samples. A 4-h playback track was constructed by concatenating randomly created stretches, providing a regular pace of 48 boat passes.

### Playback treatments

Using R (version 3.1.2) and the Seewave package (version 2.0.1)^47^, a high-pass filter was applied to all recordings in order to remove frequencies below 50 Hz (associated to electronic noises). Playbacks were performed through waterproof earphones (Enduro Sport Earphones, sensitivity −102 dB SPL/mW, frequency response 20 Hz–20 kHz, KitSound, Christchurch, Dorset, UK), connected to mp3 players (MPUB330, frequency response 20 Hz–20 kHz, Mpman, Brussels, Belgium) and to a power amplifier (F-102, 2 channel PA, Formula). Playing sounds in experimental setups can alter the characteristics of the original recordings. The analysis of the spectra of the different stimuli depicted important original characteristics preserved in playbacks. Differences between MPA, non-MPA and boat sounds detected in the field were still present (Fig. 2).

### Choice experiments

Choice experiments were performed in an acoustically insulated room in cross-shaped choice chambers modified from Lecchini *et al*.^48^ (Fig. 3). The ends of two of the four arms of each choice chamber were randomly selected to include live CCA with working underwater speakers placed behind them. The remaining two arms ended with bleached dead CCA with non-working underwater speakers behind the substrate. Chambers were made of 2.5 cm diameter PVC pipes. A central cross was fused with four 20 cm-long tubes, each one closed by an elbow oriented upright at the end. Four chambers were built and placed on separated Styrofoam panels in order to reduce the transmission of vibrations between each setup. According to the number of planulae available, between 2 to 4 randomly selected sound treatments were tested per coral species and per day (only one sound treatment per day). A total of 13 replicates were obtained for control and boat sound treatments, 12 replicates were obtained for Tetaiuo, Gendron, Tiahura, Papetoai, Temae and Nuarei treatments. For *A. cytherea*, 8 replicates were obtained for each sound treatment. Each chamber was filled with 200 mL of seawater, with no water flow. 1 g of live CCA (2 × 2 cm chip) was placed at the end of 2 elbows, and the 2 other opposite elbows contained 1 g of bleached dead CCA (2 × 2 cm chip). The use of dead CCA as alternative substrate choice was to help distinguish between the biological and physical properties of the CCA as well as volume available for swimming. Note that preliminary trials with bleached dead CCA vs no CCA in the same experimental chambers did not show a repulsive effect of dead CCA (see Supplementary Fig. S1). The positions of the live CCA pieces were randomly changed between trials. As the present experiment intends to test the effect of sound on the settlement of planulae and their natural preferential attraction toward live CCAs^8–10^, earphones were placed underwater behind all CCA pieces, facing towards the centre of the chamber. Earphones behind the dead CCA pieces were off and did not produce any sound. Only one sound was played at a time in each chamber. Sounds were only played behind the live CCA to focus the study on the cumulative impact of chemical and acoustic cues which will occur *in situ*. Playbacks from the selected sound treatments were recorded in the middle of the chambers to ensure the same sound pressure level as recorded *in situ*.

Forty coral planulae of *P. damicornis* or *A. cytherea* were introduced at t = 0 in the centre of each chamber. All planulae were tested only once and used within 12 h following their release from the parental colonies. The numbers of planulae in each arm were counted every 60 min for 4 h using an ultraviolet lamp. This observation method enhances the detection of planulae through the excitation of their green fluorescent proteins^49,50^. As initial coral movement patterns did vary over time (*i.e*., observations every 60 min – see Supplementary Figs S2 and S3), the analysis was conducted using the number of planulae present within a distance of 2/3 of each arm length from CCA chips after 4 hours in the chamber (t = 4 h). The position of the larvae after 4 h of time was considered the definitive choice. Preliminary tests allowed to define the maximum duration at 4 h in order for coral larvae to have enough time to move towards live or dead CCA and also to play all the recording of 5 minutes per hour over a 48-hr period (see also results in Supplementary Fig. S2).

Normality and homoscedasticity of the data were verified using Shapiro-Wilk’s test and Bartlett’s test, respectively. For experiments on *P. damicornis*, a two way-ANOVA with sound treatments and sound types was used to compare larval distribution (*i.e*. number of coral planulae) in the live CCA and in the dead CCA arms. Factor 1 (type of playback) has 4 levels (control, MPA, non-MPA and boat), factor 2 (sound type) has 8 levels (control, Tetaiuo, Gendron, Tiahura, Papetoai, Nuarei, Temae and boat). The analysis were followed by Tukey’s HSD post-hoc tests for pairwise comparisons in order to examine differences between playbacks and sound types. For experiments on *A. cytherea*, since only one MPA and one non-MPA sounds were used, effects of sound types on larval distribution (*i.e*. number of coral planulae) in the live CCA and in the dead CCA arms were analysed using a one-way ANOVA with sound type as fixed factor. Tukey’s HSD post-hoc tests were subsequently used to identify differences between treatments.

Lastly, preliminary trials with live CCA with ambient noise with no boat noise, recorded in Opunohu bay vs. dead CCA in the same experimental chambers demonstrated no repulsive effect of ambient noise (see Supplementary Fig. S4).

All analyses were two-tailed, at an alpha level of 0.05 and carried out in R 3.1.2 (R Core Team, 2014) using customized scripts.

### Data availability

All data generated and analysed during this study are available as Supplementary Data online.

### Ethical approval

All the experiments were approved by the CRIOBE-IRCP animal ethics committee and performed in accordance with the guidelines of the French Polynesia committee for publication and animal ethics.

## Electronic supplementary material

Supplementary Figures

Supplementary Data
