# Supplementary material for: Boat noise prevents soundscape-based habitat selection by coral planulae
Source: Sci Rep. 2018 Jun 18;8:9283. doi: 10.1038/s41598-018-27674-w (PMC6006332; doi:10.1038/s41598-018-27674-w)

SUPPLEMENTARY INFORMATION

Boat noise prevents soundscape-based habitat selection by coral planulae

David Lecchini, Frédéric Bertucci, Camille Gache, Adam Khalife, Marc Besson, Natacha Roux, Cecile Berthe, Shubha Singh, Eric Parmentier, Maggy M. Nugues, Rohan M. Brooker, Danielle L. Dixson and Laetitia Hédouin

Supplementary Figure S1. Distribution of coral planulae (% ± SE) in the choice chambers for the preliminary trials with bleached dead CCA vs no CCA (results at t = 4h; 12 replicates). The data showed no significant repulsion of coral planulae by dead.

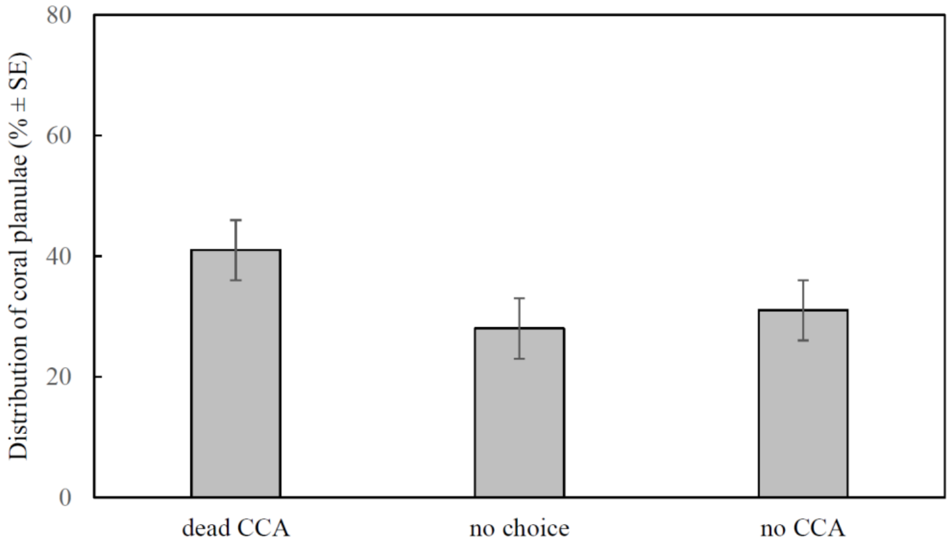

Supplementary Figure S2. Distribution of *P. damicornis* planulae (mean ± SE) in the choice chambers over the duration of the experiment for the different sound treatments. Planulae in live CCA arms are in blue, in dead CCA arms are in red and in the central ‘no-choice’ compartment are in dashed grey. No noise (control) (n = 13), Tetaiuo MPA sounds (n = 12) / Gendron non-MPA sounds (n = 12), Tiahura MPA sounds (n = 12) / Papetoai non-MPA sounds (n = 12), Nuarei MPA sounds (n = 12) / Temae non- MPA sounds (n=12) and boat noise (n = 13).

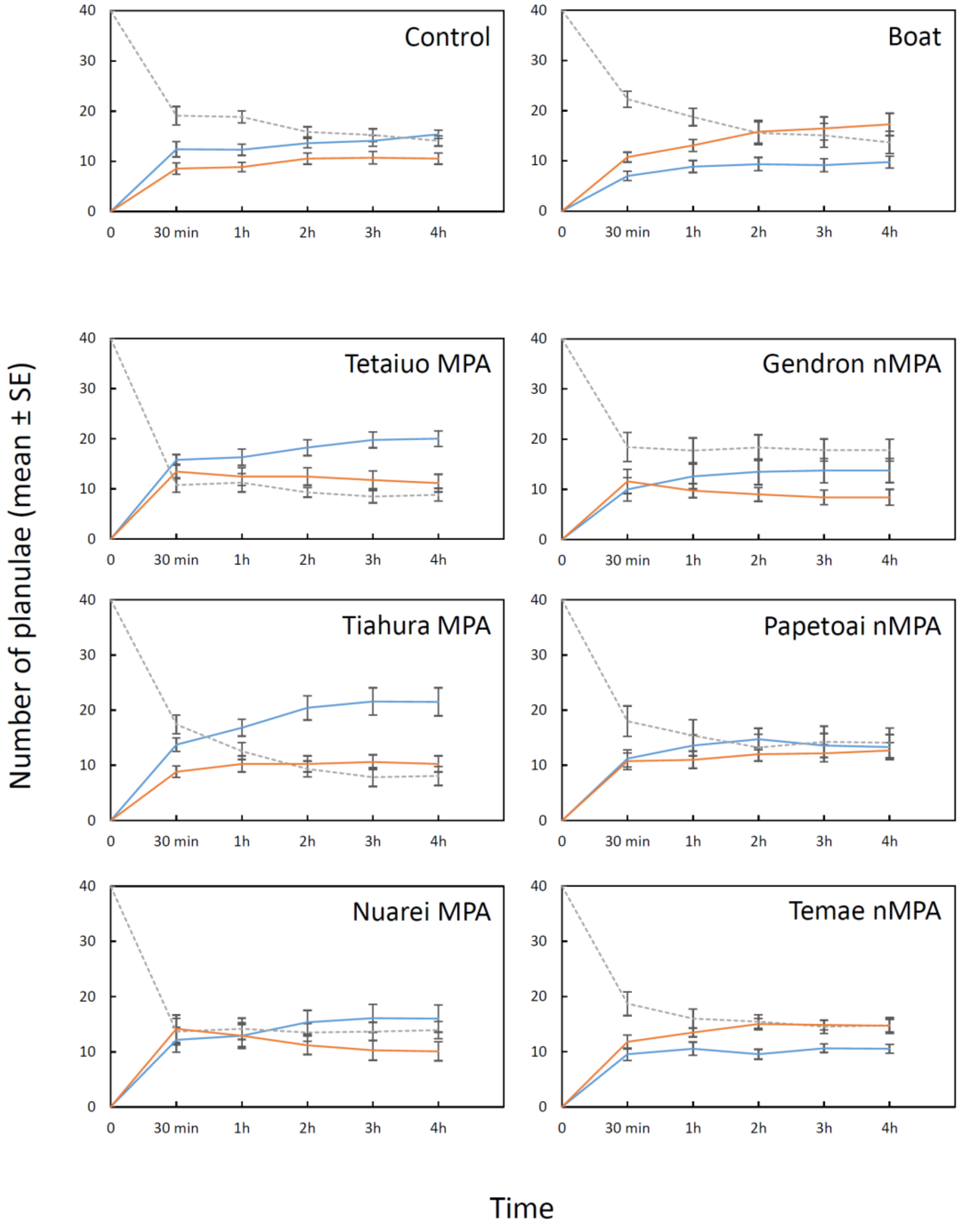

Supplementary Figure S3. Distribution of *A. cytherea* planulae (mean ± SE) in the choice chambers over the duration of the experiment for the different sound treatments. Planulae in live CCA arms are in blue, in dead CCA arms are in red and in the central ‘no-choice’ compartment are in dashed grey. Control (n = 8), boat noise (n = 8), Tiahura MPA sound (n = 8) and Papetoai non-MPA sound (n = 8).

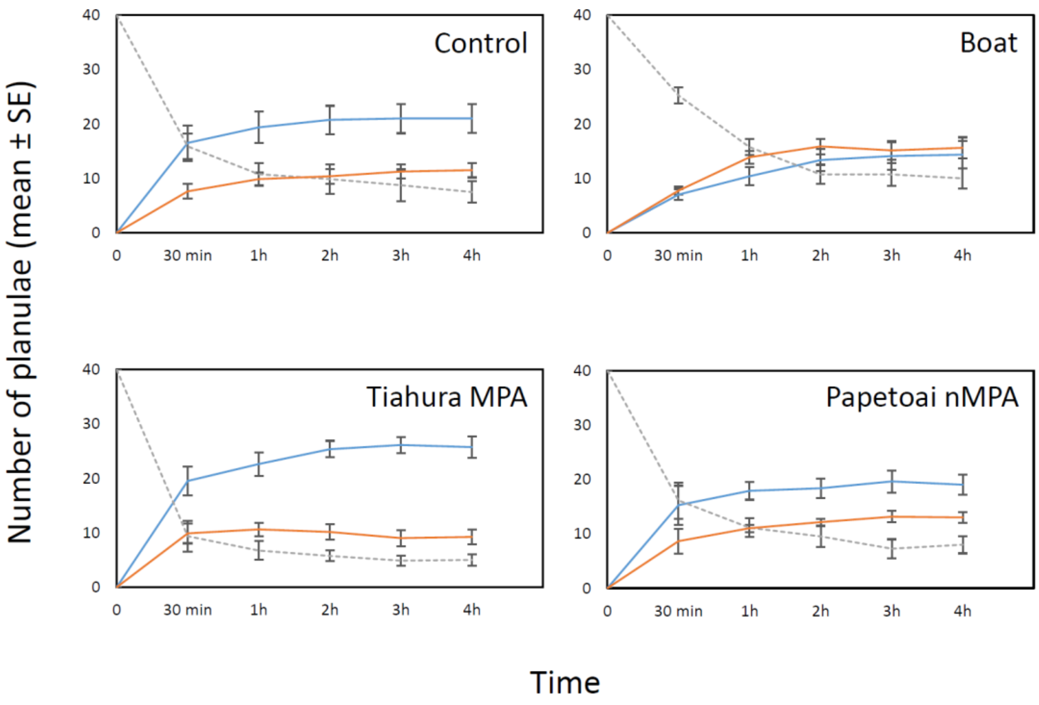

Supplementary Figure S4. Distribution of coral planulae (% ± SE) in the choice chambers for the preliminary trials with live CCA with ambient noise (Opunohu bay) vs. dead CCA (results at t = 4h; 8 replicates). The data showed no significant repulsion or attraction of ambient noise.

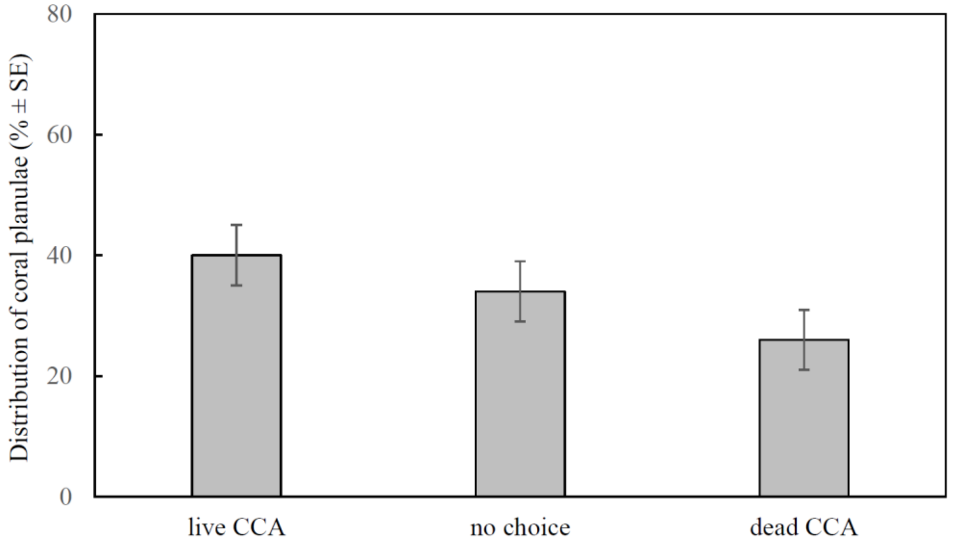

Supplement: Supplementary file 1 — Supplementary Figures [file 41598_2018_27674_MOESM1_ESM.pdf]
